# Randomisierte klinische Studie zur Kompressionstherapie der Unterschenkel bei Patienten mit Psoriasis

**DOI:** 10.1007/s00105-023-05155-0

**Published:** 2023-05-09

**Authors:** Frederik Krefting, Stefanie Hölsken, Maurice Moelleken, Joachim Dissemond, Wiebke Sondermann

**Affiliations:** 1 Klinik für Dermatologie, Venerologie und Allergologie, Universität Duisburg-Essen, Hufelandstr. 55, 45147 Essen, Deutschland; 2 Institut für Medizinische Psychologie und Verhaltensimmunbiologie, Universitätsklinikum Essen, Universität Duisburg-Essen, Essen, Deutschland

**Keywords:** Psoriasis, Unterschenkel, Kompressionstherapie, Ödeme, Köbner-Phänomen, Psoriasis, Lower extremity, Compression therapy, Edema, Koebner phenomenon

## Abstract

**Hintergrund:**

Psoriasisplaques an den Unterschenkeln zeigen sich oftmals besonders therapierefraktär. Eine Kompressionstherapie könnte an dieser Lokalisation möglicherweise eine sinnvolle ergänzende Therapiemaßnahme darstellen. Jedoch bestehen oft Bedenken, dass ein Köbner-Phänomen zu einer Verschlechterung des Hautbefundes führen könnte. Daher sollten in dieser Studie die Effekte einer Kompressionstherapie auf Psoriasisplaques bei gleichzeitig bestehenden Ödemen an den Unterschenkeln untersucht werden.

**Patienten und Methoden:**

Es erfolgte ein Halbseitenversuch bei dem zusätzlich zum „standard of care“ eine 4‑wöchige Kompressionstherapie durchgeführt wurde. Der primäre Endpunkt war das klinische Ansprechen der Psoriasis-Plaques an den Unterschenkeln im Seitenvergleich gemessen mittels Lesion Severity Score (LSS) und der lokal betroffenen Körperoberfläche in Woche 4 im Vergleich zum Ausgangsbefund. Sekundäre Endpunkte bezogen sich auf „patient-reported outcomes“.

**Ergebnisse:**

In die finale Analyse gingen die Daten von 30 Patienten ein. Die mittleren LSS-Befunde sowie die subjektiven Schmerzen der Patienten zeigten in der rein deskriptiven Auswertung eine geringgradig stärkere Verbesserung im Bereich des komprimierten im Vergleich zum nicht komprimierten Unterschenkel. Es ergaben sich keine Hinweise auf ein Köbner-Phänomen.

**Diskussion:**

Es handelt sich um die erste klinische Studie, die systematisch den Einfluss einer Kompressionstherapie auf Psoriasisplaques untersuchte. Im Untersuchungszeitraum von 4 Wochen ergaben sich zwar keine signifikanten Besserungen der Psoriasisplaques, allerdings auch keine Hinweise für eine Verschlechterung des Hautbefundes. Folglich kann eine antiödematöse Kompressionstherapie bei Patienten mit Psoriasis unter Beachtung der grundlegenden Kontraindikationen ohne Komplikationen durchgeführt werden.

## Abstract (en)

**Background:**

Psoriatic plaques at the distal lower extremities are notoriously treatment resistant. Medical compression therapy could potentially be a useful supplementary therapeutic measure at this site. However, there is concern that the Koebner phenomenon may cause a worsening of the skin condition. Therefore, the purpose of this study was to investigate the effects of compression therapy on psoriatic plaques in the presence of coexisting edema of the lower legs.

**Patients and methods:**

Compression therapy was performed in addition to standard of care on one lower leg for 4 weeks (half-side test) in patients with psoriatic plaques and edema on both lower legs. The primary endpoint of the study was clinical response of the psoriatic plaques on the lower legs measured with the lesion severity score (LSS) and the locally affected body surface area in a side-by-side comparison at week 4 compared with baseline. Secondary endpoints were related to patient-reported outcomes.

**Results:**

Data from 30 patients were included in the analysis. In the descriptive analysis, the mean LSS results and the subjective pain reported by the patients showed a slightly greater improvement on the compressed lower leg compared with the non-compressed lower leg. None of the patients showed evidence of the Koebner phenomenon induced by compression therapy.

**Conclusion:**

This is the first clinical study that systematically investigated the impact of compression therapy on psoriatic plaques. During the study period of 4 weeks, there was no significant improvement in psoriatic plaques; however, there was also no evidence of worsening of the skin condition. Thus, anti-edematous compression therapy can be performed in psoriasis patients without causing complications if basic contraindications are considered.

Psoriasisplaques an den Unterschenkeln zeigen sich bei vielen Patienten besonders therapierefraktär und werden häufig von Ödemen begleitet. Somit könnte eine medizinische Kompressionstherapie eine sinnvolle therapeutische Ergänzung zu den aktuellen Behandlungskonzepten darstellen. Allerdings bestehen oft Bedenken, dass durch die Kompressionstherapie ein Köbner-Phänomen induziert und eine Verschlechterung der Psoriasis ausgelöst werden könnte. In der hier gezeigten klinischen Studie sollten daher die Effekte einer Kompressionstherapie auf den Verlauf der Psoriasis untersucht werden.

## Hintergrund und Fragestellung

Die Psoriasis ist eine genetisch determinierte, chronisch entzündliche, immunvermittelte Systemerkrankung, die durch multifaktorielle extrinsische oder intrinsische Einflüsse provoziert werden kann und global Menschen jeden Alters betrifft. In Deutschland leiden ungefähr 2 Mio. Menschen an einer Psoriasis [1]. Die Erkrankung verursacht für die Betroffenen große körperliche, emotionale und soziale Belastungen und geht für das Gesundheitssystem mit erheblichen Kosten einher [27].

Die chronische Inflammation bei der Psoriasis kommt durch ein komplexes Zusammenspiel von Genen, Umweltfaktoren und dem Immunsystem zustande. Triggerfaktoren, die zu einer Exazerbation führen können, sind u. a. mechanische Reize, pharmakologische Trigger, Infekte und psychologische Faktoren [3, 6, 8, 15, 16]. Das Verständnis der Pathophysiologie der Psoriasis hat in den vergangenen Jahren enorm zugenommen. Zentrale proinflammatorische Zytokine bei der psoriatischen Entzündungsreaktion sind Tumor-Nekrose-Faktor-alpha (TNF-α), Interleukin(IL)-23 und IL-17, die heute durch Biologikatherapien oder „small molecules“ zielgerichtet adressiert werden können [21, 23].

Die Prädilektionsstellen der bei der Psoriasis typischerweise auftretenden, erythrosquamösen Plaques befinden sich insbesondere an den Streckseiten der Extremitäten. Aus dem klinischen Alltag ist bekannt, dass sich Psoriasisplaques an den Unterschenkeln im Vergleich zu anderen betroffenen Körperstellen oftmals besonders therapierefraktär und hartnäckig zeigen. So werteten z. B. Hjuler et al. 146 Therapieverläufe von Psoriasispatienten unter Biologikatherapien aus und stellten fest, dass sich die therapierefraktärsten Plaques bevorzugt an den ventralen und dorsalen Unterschenkeln befanden [7]. Das Vorliegen von Psoriasisplaques an gut sichtbaren Arealen ist aufgrund der damit einhergehenden verstärkten sozialen Stigmatisierung für die Patienten oft besonders belastend. Die Unterschenkel können v. a. im Sommer, aber auch z. B. bei sportlichen Aktivitäten sichtbar sein, sodass eine Abheilung an diesen Körperstellen für die Patienten sehr wichtig ist. Die Genese der erschwerten Abheilung von Psoriasisplaques an den Unterschenkeln ist nicht genau bekannt. Über die Inflammation der Haut könnte es an den Unterschenkeln zu einer vermehrten Ödemneigung kommen, sodass die Abheilung der chronisch persistierenden Entzündungsreaktion eventuell stasebedingt verlangsamt abläuft.

Diese Hypothese wirft die Frage nach einer möglicherweise positiven Wirkung der medizinischen Kompressionstherapie auf. Die aktuelle deutsche Leitlinie der Deutschen Gesellschaft für Phlebologie (DGP) zur medizinischen Kompressionstherapie der Extremitäten nennt das Vorliegen einer entzündlichen Dermatose an den Unterschenkeln unter den sog. „anderen Indikationen“ – auch wenn genauere Erläuterungen hierzu nicht weiter ausgeführt werden [18]. Es gibt zunehmend wissenschaftliche Belege für die positive Wirkung einer Kompressionstherapie auf entzündliche Prozesse in der Haut. So wurde z. B. ein signifikant abnehmender Spiegel proinflammatorischer Zytokine wie Interferon‑γ in der Haut von Patienten mit Ulcus cruris venosum im Verlauf einer 4‑wöchigen Kompressionstherapie nachgewiesen [2, 11]. Zudem liegen erste kasuistische Beschreibungen positiver Verläufe von an den Unterschenkeln lokalisierten Psoriasisplaques unter einer Kompressionstherapie vor [11, 22, 25]. Ergebnisse klinischer Studien zu dem Einfluss der Kompressionstherapie auf Psoriasisplaques an den Unterschenkeln existieren jedoch bislang nicht. Bedenken gegenüber einer Kompressionstherapie bei Patienten mit Psoriasis bestehen oft, da es durch die mechanische Reibung zu einem Köbner-Phänomen mit dem Neuauftreten oder einer Exazerbation von bereits bestehenden Plaques kommen könnte. Das Auftreten eines pathophysiologisch noch nicht vollständig geklärten Köbner-Phänomens bei Psoriasispatienten wird mit einer Häufigkeit von 11–75 % angegeben [4].

Das Ziel der vorliegenden klinischen Studie bestand daher darin, mithilfe eines Halbseitenversuchs die Fragestellung zu untersuchen, inwiefern durch das Tragen einer Kompressionstherapie die Abheilung von Psoriasisplaques an den Unterschenkeln bei vorliegenden Ödemen beeinflusst werden kann.

## Patienten und Methoden

### Ein- und Ausschlusskriterien

Innerhalb dieser prospektiven, monozentrischen, randomisierten, untersucherverblindeten, interventionellen Studie wurden Patienten mit Psoriasisplaques und Ödemen an beiden Unterschenkeln einem Halbseitenversuch im Sinne eines Within-subject-Designs unterzogen (Tab. 1). Eine 4‑wöchige Kompressionstherapie wurde an einem Unterschenkel durchgeführt.

**Table 1:** 

*Einschlusskriterien*
Alter ≥ 18 Jahre
Psoriasis seit mindestens 6 Monaten
Ödeme an beiden Unterschenkeln
Psoriasisplaques an beiden Unterschenkeln
Fähigkeit Kompressionsstrümpfe selbstständig an- und ausziehen zu können
*Ausschlusskriterien*
Unzureichende Kommunikationsfähigkeit in der deutschen Sprache bzw. mangelndes Sprachverständnis der deutschen Sprache oder Demenz oder (funktioneller) Analphabetismus
Mangelnde oder fehlende Einwilligungsfähigkeit des Patienten
Dekompensierte Herzinsuffizienz
Fortgeschrittene Polyneuropathie
Schwangerschaft und Stillzeit
Kritische Ischämie (Knöchel-Arm-Druck-Index < 0,5 oder absoluter Druck < 60 mm Hg)
Bekannte chronisch venöse Insuffizienz oder andere Erkrankungen wegen derer bereits eine Kompressionstherapie getragen wird
Floride Wunde an den unteren Extremitäten
Adipositas per magna (BMI > 40)

*mm Hg* Millimeter-Quecksilbersäule, *BMI* Body Mass Index

### Studienablauf

Zu Beginn der Studie (Visite 1) wurden der Schweregrad der Psoriasis insgesamt mittels Psoriasis Area and Severity Index (PASI) und die betroffene Körperoberfläche in Prozent (KOF) ermittelt. Zudem wurde die Ausprägung von Psoriasisplaques an beiden Unterschenkeln bestimmt. Die zu vergleichenden Psoriasisplaques der Unterschenkel wurden durch die lokal betroffene KOF sowie den Läsionsschweregrad (Lesion Severity Score [LSS]) bewertet, der analog zum PASI das vorliegende Erythem, die Infiltration und die Schuppung auf einer Skala von 0 (keine) bis 4 (sehr stark) einteilt und als Summe der verschiedenen Punktwerte der einzelnen Domänen gebildet wird [17]. Bei Schmerzen wurden diese als maximale Schmerzen der Unterschenkel in den letzten 24 h mit einer visuellen Analogskala (VAS) getrennt nach linkem und rechtem Bein beschrieben. Zudem wurde der Dermatology Life Quality Index (DLQI) zum Start der Studie zur Bewertung der hautbezogenen Lebensqualität erhoben. Schließlich wurden die Beinumfänge an 2 definierten Messpunkten (20 cm und 35 cm Abstand senkrecht zum Boden ohne Schuhwerk) zu Beginn der Studie gemessen, um den Verlauf der Ödeme zu dokumentieren.

Mittels Messung des Knöchel-Arm-Druck-Index (KADI) wurde eine fortgeschrittene periphere arterielle Verschlusskrankheit ausgeschlossen. An einem Unterschenkel erhielten die Studienteilnehmer den rundgestrickten medizinischen Kompressionsstrumpf „mediven® plus“ nach Maß inklusive Wechselversorgung (Länge A–D, Kompressionsklasse II, 23–32 mm Hg), der von der medi GmbH & Co. KG, Firmensitz Bayreuth, Deutschland, zur Verfügung gestellt wurde. Die Auswahl der Unterschenkel, die mit einem Kompressionsstrumpf versorgt wurden, erfolgte nach einem zuvor festgelegten System, bei dem sich rechts und links im Verhältnis 1:1 abwechselten. Die Patienten erhielten die einseitige Kompressionstherapie zusätzlich zum „standard of care“ (SoC). Dies bedeutet, dass sowohl topische als auch systemische Psoriasistherapien neu eingeleitet bzw. fortgeführt werden konnten. Die Kompressionstherapie sollte während des 4‑wöchigen Studienzeitraums täglich mindestens für 8 h getragen werden. Zur Dokumentation der Tragezeiten wurde ein Tagebuch ausgehändigt, in welchem seitens der Patienten auch Kommentare eingefügt werden konnten.

Die Beurteilung des Ausprägungsgrades der Psoriasisplaques an den Unterschenkeln mittels LSS und KOF sowie die Bestimmung des Gesamt-PASI und der Gesamt-KOF erfolgte nach 4 Wochen (Visite 2) durch einen verblindeten Studienarzt, der keine Kenntnis hatte, welches Bein einer Kompressionstherapie unterzogen worden war. Damit der verblindete Untersucher nicht anhand von Abdrücken des Gummibündchens erkennen konnte, an welchem Bein der Kompressionsstrumpf getragen wurde, wurde der Patient gebeten, seinen Kompressionsstrumpf in den 12 h vor Visite 2 nicht zu tragen. Bei Visite 2 wurden zudem erneut Schmerzen im Seitenvergleich mittels VAS abgefragt, der DLQI bestimmt sowie die Beinumfänge erhoben. Am Ende der Studie wurden die Patienten zudem mithilfe eines Fragebogens bezüglich ihrer Zufriedenheit und den Erfahrungen mit der Kompressionstherapie befragt.

### Endpunkte

Der primäre Endpunkt der Studie war das klinische Ansprechen der Psoriasisplaques an den Unterschenkeln im Seitenvergleich (SoC vs. SoC + Kompressionstherapie) gemessen mittels LSS sowie KOF in Woche 4 im Vergleich zum Ausgangsbefund, sodass ein möglicherweise auftretendes Köbner-Phänomen oder Verbesserungen der Hautveränderungen objektiviert werden konnten. Sekundäre Endpunkte waren Schmerzen anhand der VAS, Entwicklung der Beinumfänge sowie Zufriedenheit und Erfahrung mit der Kompressionstherapie aus Patientensicht in Woche 4 im Vergleich zum Ausgangsbefund.

### Auswertung, Patientenrechte und Studienzeitraum

Die Durchführung der Studie wurde von der Ethikkommission der Universität Duisburg-Essen genehmigt (18-8366-BO). Alle beschriebenen Untersuchungen wurden in Einklang mit nationalem Recht sowie gemäß der Deklaration von Helsinki durchgeführt. Von allen beteiligten Patienten wurde vor Studienbeginn eine schriftliche Einverständniserklärung eingeholt.

Die pseudonymisierte Datensammlung und -auswertung erfolgte mithilfe der Statistik-Software SPSS Statistics Version 27 (Fa. IBM, Armonk, NY, USA).

Für die Auswertung der Hauptfragestellungen wurden 3 messwiederholte ANOVAs gerechnet: je eine für die abhängigen Variablen LSS, KOF und Schmerzen. Die messwiederholten Inner-Subjekt-Faktoren waren der Messzeitpunkt (Baseline und nach 4 Wochen) und der Unterschenkel (komprimiert und nicht komprimiert).

Für die Subgruppenanalysen zur Bestimmung des Einflusses einer Systemtherapie, des Umfangs des komprimierten Unterschenkels zur Baseline (separat für 20 cm und 35 cm) sowie der Umfangsreduktion (ebenfalls für 20 cm und 35 cm) wurde für jeden dieser Zwischen-Subjekt-Faktoren sowie pro abhängiger Variable (LSS, KOF, Schmerzen) eine gemischte ANOVA gerechnet. Für die kontinuierlichen Variablen Umfang und Umfangsreduktion wurde dabei ein Median-Split durchgeführt, um einen Faktor mit 2 möglichen Ausprägungen (stärkere/geringere Ödeme zu Baseline und stärkere/geringere Ödemreduktion nach Woche 4) zu generieren. Grafiken wurden in Python (Python Software Foundation, Version 3.6, Wilmington, Delaware, Vereinigte Staaten) erstellt.

## Ergebnisse

### Demografie

Zwischen April 2019 und April 2022 wurden insgesamt 35 Patienten in die Studie eingeschlossen. Da 5 Patienten nicht zur Visite 2 erschienen, wurden diese Datensätze zensiert und als „lost to follow-up“ gewertet, sodass Datensätze von insgesamt 30 Patienten (*n* = 21 männlich [70 %] und *n* = 9 weiblich [30 %]) zur vollständigen Analyse vorlagen. Die Patienten waren im Durchschnitt 52,8 Jahre alt und wiesen einen mittleren Body-Mass-Index (BMI) von 28,7 kg/m^2^ auf. Die häufigsten Komorbiditäten waren arterielle Hypertonie, Adipositas, Nikotinabusus und Diabetes mellitus. Insgesamt 83,3 % der Patienten wandten an den Unterschenkeln eine Lokaltherapie an, und 76,7 % aller Patienten waren bereits unter einer Systemtherapie bzw. starteten diese zu Studienbeginn (Tab. 2).

**Table 2:** 

	Gesamtstichprobe *N* (%)
**Patientenanzahl**	30 (100,0)
**Mittleres Alter in Jahren (Min.–Max.)**	52,8 (19–77)
**Mittlerer Body-Mass-Index in kg/m**^**2**^** (Min.–Max.)**	28,7 (20,1–39,9)
**Geschlecht**	
*Weiblich*	9 (30,0)
*Männlich*	21 (70,0)
**Häufigste Komorbiditäten**	
*Arterielle Hypertonie*	13 (43,3)
*Adipositas*	10 (33,3)
*Nikotinabusus*	10 (33,3)
*Diabetes mellitus*	3 (10,0)
**Verwendete Lokaltherapien**	
*Nein*	5 (16,7)
*Ja*	25 (83,3)
Calcipotriol in Kombination mit Betamethason	11 (36,7)
Calcipotriol	6 (20,0)
Glukokortikoid	2 (6,7)
Hautpflegeprodukte	6 (20,0)
**Verwendete Systemtherapien**	
*Nein*	7 (23,3)
*Ja*	23 (76,7)
Biologika	12 (40,0)
– TNF-α-Inhibitor	1 (3,3)
– Certolizumab	1 (3,3)
– Interleukin-12/23-Inhibitor	1 (3,3)
– Ustekinumab	1 (3,3)
– Interleukin-17A-Inhibitor	4 (13,3)
– Secukinumab	1 (3,3)
– Ixekizumab	3 (10,0)
– Interleukin-23-Inhibitor	6 (20,0)
– Tildrakizumab	2 (6,7)
– Risankizumab	2 (6,7)
– Guselkumab	2 (6,7)
Konventionelle Systemtherapie	11 (36,6)
– Methotrexat	7 (23,3)
– Dimethylfumarat	4 (13,3)

*Min.* Mindestens, *Max.* Maximal, *TNF-α* Tumornekrosefaktor-α

### Verlauf von Gesamt-PASI, Gesamt-KOF und DLQI

Im Verlauf der Studie zeigten sich sowohl der Gesamt-PASI und die Gesamt-KOF sowie der DLQI regredient (Tab. 3).

**Table 3:** 

Eigenschaften (*n* = 30)	Psoriasis Area and Severity Index (PASI)	Betroffene Gesamtkörperoberfläche (KOF) in %	Dermatology Life Quality Index (DLQI)
Mittelwert Visite 1	9,70	18,98	8,70
Std.-Abweichung Visite 1	6,26	15,92	5,44
Mittelwert Visite 2	8,37	14,49	6,13
Std.-Abweichung Visite 2	7,13	13,49	6,10
Absolute Veränderung	−1,33	−4,49	−2,57
*p*-Wert	0,45	0,24	0,09

*PASI* Psoriasis Area and Severity Index; *KOF* Körperoberfläche; *DLQI* Dermatology Life Quality Index; *Std.-Abweichung* Standardabweichung

### Auswirkungen der Kompressionstherapie

**Figure 1:**
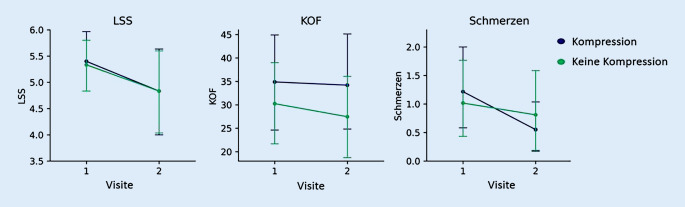

Die Mittelwerte der Parameter LSS, KOF der Unterschenkel sowie die mittels VAS erhobenen Schmerzen zeigten sowohl bei der Bewertung des komprimierten Unterschenkels als auch des nicht komprimierten Unterschenkels eine Verbesserung zwischen Visite 1 und 2. Im Vergleich der mittleren LSS-Befunde zwischen komprimiertem und nicht komprimiertem Unterschenkel war eine geringe, deskriptive Überlegenheit des komprimierten Unterschenkels (absolute Veränderung: −0,56 [komprimiert] vs. −0,50 [nicht komprimiert]) nachweisbar. Ähnlich zeigte sich der Vergleich der mittleren VAS der Schmerzen, der ebenfalls zugunsten des komprimierten Unterschenkels ausfiel (absolute Veränderung: −0,67 [komprimiert] vs. −0,21 [nicht komprimiert]). Die mittlere KOF wies im Laufe der Studie eine etwas größere Abnahme beim nicht komprimierten Unterschenkel auf (absolute Veränderung: −0,68 [komprimiert] vs. −2,78 [nicht komprimiert]) (Abb. 1 und 2). Statistisch signifikante Unterschiede fanden sich im Vergleich der Unterschenkel in den genannten Parametern nicht (Tab. 4).

**Figure 2:**
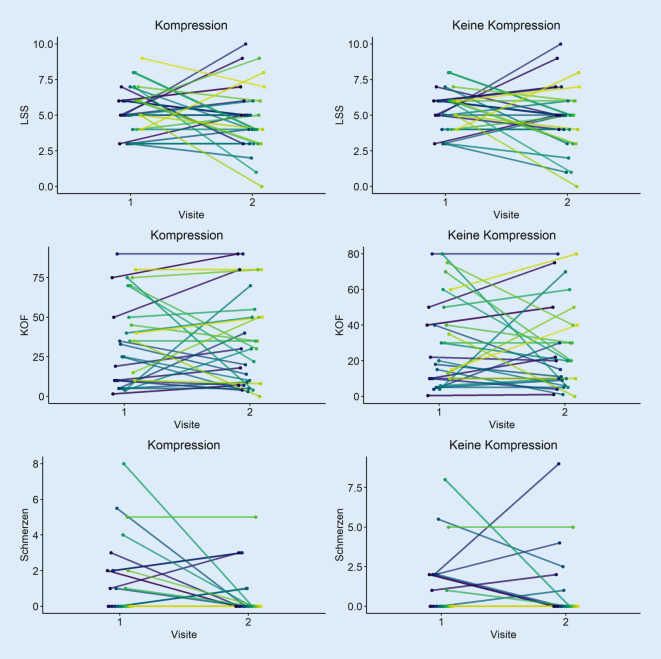

**Table 4:** 

Eigenschaften (*n* = 30)	Lesion Severity Score (LSS)		Betroffene Körperoberfläche der Unterschenkel (KOF) in %		Maximale Schmerzen der Unterschenkel anhand der visuellen Analogskala (VAS)	
	Kompression	Keine Kompression	Kompression	Keine Kompression	Kompression	Keine Kompression
Mittelwert Visite 1	5,40	5,33	34,88	30,25	1,22	1,02
Std.-Abweichung Visite 1	1,61	1,40	27,55	25,08	2,02	1,95
Mittelwert Visite 2	4,83	4,83	34,20	27,47	0,55	0,81
Std.-Abweichung Visite 2	2,29	2,25	28,95	24,99	1,27	2,02
Absolute Veränderung	−0,56	−0,50	−0,68	−2,78	−0,67	−0,21
*p*-Wert	0,70		0,39		0,33	

*Std.-Abweichung* Standardabweichung

Zusätzlich wurden Subgruppenanalysen der primären Endpunkte in Bezug auf eine erhaltene Systemtherapie, Patienten mit stärkeren Ödemen im Vergleich zu geringeren Ödemen bei Studienstart sowie Patienten mit stärkerer Ödemreduktion im Vergleich zu einer geringeren Ödemreduktion durch die Kompressionstherapie im Verlauf durchgeführt. Statistisch signifikante Unterschiede fanden sich in den Subgruppenanalysen nicht.

### Tragedauer und Komfort der Kompressionstherapie

Insgesamt wurde der Komfort der Kompressionstherapie positiv bewertet (Mittelwert nach Schulnoten 2,07, Standardabweichung 0,74). Mehr als die Hälfte der Patienten beschrieb das Trageempfinden als angenehm (43,3 %) bzw. sehr angenehm (10,0 %). Lediglich 16,7 % der befragten Patienten empfanden das Tragen der Kompressionstherapie als eher unangenehm. Von den Befragten vergaben 76,7 % dementsprechend mindestens die Schulnote „gut“ in Bezug auf den Tragekomfort. Schnürfurchen und Druckstellen blieben in den allermeisten Fällen aus. Die Auswertung der Patiententagebücher ergab, dass bei 22 Patienten (73,3 %) keinerlei Trageunterbrechungen auftraten. Die 8 Patienten, die Trageunterbrechungen angaben (26,7 %), berichteten in keinem Fall eine Trageunterbrechung von mehr als 3 Tagen (Tab. 5).

**Table 5:** 

Tragedauer und Komfort der Kompressionstherapie entsprechend der Tagebucheinträge		
**–**	Häufigkeit	Prozentuale Anteile (*n* = 30)
*Trageunterbrechung* *(n* *=* *30)*		
Ja	8	26,7
Nein	22	73,3
*Trageunterbrechung* *(n* *=* *8)*		
≤ 1 Tag	3	10,0
1 bis 3 Tage	5	16,7
≥ 3 Tage	0	0
*Schnürfurchen* *(n* *=* *30)*		
Keine	27	90,0
Leicht	2	6,7
Mittel	1	3,3
Schwer	0	0
*Druckstellen* *(n* *=* *30)*		
Keine	25	83,3
Leicht	4	13,3
Mittel	1	3,3
Schwer	0	0
*Trageempfinden* *(n* *=* *30)*		
Sehr angenehm	3	10,0
Angenehm	13	43,3
Eher angenehm	9	30,0
Eher unangenehm	5	16,7
Unangenehm	0	0
Sehr unangenehm	0	0
*Tragekomfort* *(n* *=* *30)*		
Sehr gut	6	20,0
Gut	17	56,7
Befriedigend	6	20,0
Ausreichend	1	3,3
Mangelhaft	0	0
Ungenügend	0	0

## Diskussion

In der vorliegenden Arbeit wurden mithilfe eines Halbseitenversuchs über 4 Wochen die Effekte einer Kompressionstherapie auf den Verlauf von Psoriasisplaques an den Unterschenkeln bei gleichzeitig bestehenden Ödemen untersucht.

Die Ergebnisse der Studie an 30 Psoriasispatienten zeigen, dass eine Kompressionstherapie nicht zu einer Verschlechterung des Hautbefundes führte. Es ergaben sich somit keine Hinweise dafür, dass bei Psoriasispatienten mit Unterschenkelödemen durch eine Kompressionstherapie ein Köbner-Phänomen ausgelöst wird, wodurch neue Psoriasismanifestationen induziert oder zuvor bestehende Plaques aggraviert werden könnten.

Diese Erkenntnis ist für die Versorgung von Psoriasispatienten aus verschiedenen Gründen wertvoll. Zum einen ist aus der klinischen Erfahrung bekannt, dass es bei Psoriasispatienten mit großflächigen, stark inflammatorischen Psoriasisplaques an den Unterschenkeln zu ödematösen Schwellungen der Extremitäten kommen kann. Auch wenn Ergebnisse aus großen epidemiologischen Studien fehlen, ist aufgrund der sehr gut belegten internistischen Komorbiditäten der Psoriasis wie eine Nieren- oder Herzinsuffizienz von einem erhöhten Risiko für die Entstehung von Unterschenkelödemen auszugehen [10, 20, 26]. Darüber hinaus ist eine chronische venöse Insuffizienz in der Allgemeinbevölkerung weit verbreitet und stellt eine Hauptindikation für eine medizinische Kompressionstherapie dar [18, 19].

Eine antiödematöse Kompressionstherapie kann entsprechend unseren Studienergebnissen nach Ausschluss von Kontraindikationen [18] bei Psoriasispatienten also ohne Sorge vor einer Verschlechterung des Hautbefundes eingeleitet werden. Darüber hinaus könnte eine Kompressionstherapie positive Auswirkungen auf die Abheilung von Psoriasisplaques an den Unterschenkeln haben. Es zeigten sich die von den Patienten berichteten Schmerzen im Bereich der Unterschenkel an dem über 4 Wochen komprimierten Bein im Vergleich zur nicht komprimierten Gegenseite bei einem geringen Ausgangsmittelwert in etwa halbiert. Auch der Ausprägungsgrad der Hautläsionen gemessen mit dem LSS zeigte für die komprimierte Seite einen günstigeren Verlauf im Vergleich zur nicht komprimierten Seite. Für die Ausmaße der von Psoriasisplaques betroffenen Fläche war dies jedoch nicht der Fall. Statistisch signifikante Unterschiede fanden sich allerdings für keinen der bestimmten Parameter, was möglicherweise auf die geringe Fallzahl und den kurzen Beobachtungszeitraum zurückzuführen ist. Eine mögliche Erklärung für den deskriptiv verstärkten Rückgang des LSS unter der Kompressionstherapie könnte ein primäres Ansprechen der entzündlichen Prozesse auf die Kompressionstherapie sein, die im LSS anhand des Erythems gemessen werden, aber nicht Teil der Bestimmung der KOF sind.

Pathophysiologisch wäre es durchaus vorstellbar, dass eine Kompressionstherapie der Unterschenkel das Auftreten von Psoriasisplaques verhindert und die Abheilung der dort lokalisierten Psoriasisplaques fördert [7, 9]. Eine verstärkte inflammationsbedingte Ödemneigung, eine vermehrte Stase sowie der an der unteren Extremität erhöhte hydrostatische Druck könnten zu einem verzögerten oder insuffizienten Therapieansprechen beitragen. Mittels Videodermatoskopie konnte kürzlich gezeigt werden, dass der Kapillardurchmesser im Bereich von Psoriasisplaques an den unteren Extremitäten im Vergleich zu Psoriasisplaques an anderen Körperstellen vergleichsweise hoch ist und die Kapillaren eine Tendenz zu einer buschartigen Verzweigung aufweisen, was für einen erhöhten Gefäßfluss spricht [13].

Maßgeblich für den Erfolg einer Kompressionstherapie sind Compliance und Adhärenz der Patienten [5]. In der vorliegenden Studie wurde der Tragekomfort der Kompressionstherapie fast ausschließlich als angenehm bewertet, sodass die vorgesehenen Tragedauern bis auf wenige Trageunterbrechungen erreicht werden konnten. Vergleichbare Resultate zeigten sich auch z. B. bei 89 % der von Stücker et al. untersuchten 414 Patienten, die mindestens zufrieden mit der medizinischen Kompressionstherapie waren und diese durchschnittlich 10,8 h an 6,1 Tagen pro Woche trugen [24]. Da die Tragedauer in der vorliegenden Studie nicht objektiv, sondern subjektiv über ein Patiententagebuch gemessen wurde, ist kritisch anzumerken, dass die Patienten möglicherweise eine andere Tragedauer angaben, als tatsächlich stattgehabt. Studienergebnisse mit Daten aus Thermosensorenmessungen belegen, dass die von Patienten angegebenen Tragedauern häufig überschätzt werden [14, 24]. Als Treiber dieses Phänomens wird die soziale Erwünschtheit in Bezug auf ein konsequentes Tragen der medizinischen Kompressionsstrümpfe (MKS) in Fragebögen oder Interviews diskutiert [24]. Dennoch ist die insgesamt günstige Einordnung des subjektiven Tragekomforts hinsichtlich der hier verwendeten MKS als positiv anzusehen. Weitere Stärken der vorliegenden Arbeit sind das Within-subject-Design, das den Vorteil bietet, den Einfluss von z. B. Systemtherapien auf die Psoriasis bestmöglich ausschließen zu können, und der prospektive Ansatz in einem Real-World-Setting, was die Übertragbarkeit der Ergebnisse in den klinischen Alltag erleichtert.

## Limitationen

Neben der fehlenden absoluten Kontrolle über die Adhärenz der Patienten sind als Limitationen der vorliegenden Studie die mit 30 auswertbaren Patienten relativ geringe Fallzahl und der eher kurze Beobachtungszeitraum von 4 Wochen anzusehen. Es ist aus der Psoriasisbehandlung bekannt, dass es bis zu 8 Wochen dauern kann, bis unter äußerlichen Therapiemaßnahmen ein ausreichendes Ansprechen erreicht wird [12]. Eine weitere Limitation ergibt sich in Bezug auf die gemessene Ödemreduktion am komprimierten Bein. Da die Patienten zur Aufrechterhaltung der Verblindung gebeten wurden, 12 h vor der 2. Visite ihren Kompressionsstrumpf nicht mehr zu tragen, könnte die gemessene Ödemreduktion geringer als die tatsächliche ausgefallen sein.

## Fazit für die Praxis

- Es werden die Ergebnisse der ersten randomisierten klinischen Studie zur Kompressionstherapie bei Patienten mit Psoriasisplaques und Ödemen an den unteren Extremitäten vorgestellt.
- Es ergaben sich keine Hinweise dafür, dass die medizinische Kompressionstherapie bei den Patienten durch ein Köbner-Phänomen zu einer Verschlechterung der Psoriasis führt.
- Für einzelne der erhobenen Parameter, wie z. B. Schmerzen im Bereich der Unterschenkel, ergab sich eine Tendenz hin zu einem Vorteil für das komprimierte Bein.
- Eine medizinische Kompressionstherapie könnte eine sinnvolle additive therapeutische Maßnahme zu den aktuellen Behandlungskonzepten von Patienten mit Psoriasis darstellen.
- Größer angelegte klinische Studien mit längeren Behandlungsdauern sind erforderlich, um die Effekte der Kompressionstherapie auf Psoriasisplaques an den Unterschenkeln zu objektivieren.

## References

[1] Augustin M, Reich K, Glaeske G (2010). Co-morbidity and age-related prevalence of psoriasis: Analysis of health insurance data in Germany. Acta Derm Venereol.

[2] Beidler SK, Douillet CD, Berndt DF (2009). Inflammatory cytokine levels in chronic venous insufficiency ulcer tissue before and after compression therapy. J Vasc Surg.

[3] Boehncke WH, Schon MP (2015). Psoriasis. Lancet.

[4] Boyd AS, Neldner KH (1990). The isomorphic response of Koebner. Int J Dermatol.

[5] Dissemond J, Assenheimer B, Bültemann A (2016). Kompressionstherapie bei Patienten mit Ulcus cruris venosum. J Dtsch Dermatol Ges.

[6] Griffiths CE, Barker JN (2007). Pathogenesis and clinical features of psoriasis. Lancet.

[7] Hjuler KF, Iversen L, Rasmussen MK (2019). Localization of treatment-resistant areas in patients with psoriasis on biologics. Br J Dermatol.

[8] Hunter HJ, Griffiths CE, Kleyn CE (2013). Does psychosocial stress play a role in the exacerbation of psoriasis?. Br J Dermatol.

[9] Jeon C, Nakamura M (2017). “Two-step phototherapy” for treatment-resistant psoriasis on the lower extremities. J Am Acad Dermatol.

[10] Khalid U, Ahlehoff O, Gislason GH (2014). Psoriasis and risk of heart failure: a nationwide cohort study. Eur J Heart Fail.

[11] Konschake W, Valesky E, Stege H (2017). Evidenz der Kompressionstherapie. Hautarzt.

[12] Körber A, Wilsmann-Theis D, Augustin M (2019). Topische Therapie bei Psoriasis vulgaris – ein Behandlungspfad. J Dtsch Dermatol Ges.

[13] Lacarrubba F, Verzì AE, Musumeci ML (2020). High capillary diameter in psoriatic plaques of the lower legs. Br J Dermatol.

[14] Lurie F, Schwartz M (2017). Patient-centered outcomes of a dual action pneumatic compression device in comparison to compression stockings for patients with chronic venous disease. J Vasc Surg Venous Lymphat Disord.

[15] Malhotra SK, Mehta V (2008). Role of stressful life events in induction or exacerbation of psoriasis and chronic urticaria. Indian J Dermatol Venereol Leprol.

[16] Nestle FO, Kaplan DH, Barker J (2009). Psoriasis. N Engl J Med.

[17] Patel RV, Tsui CL (2011). Evaluating psoriasis: A review of the assessments most commonly used in clinical trials. Psoriasis Forum.

[18] Rabe E, Földi E, Gerlach H (2021). Medizinische Kompressionstherapie der Extremitäten mit medizinischem Kompressionsstrumpf (MKS), phlebologischem Kompressionsverband (PKV) und medizinischen adaptiven Kompressionssystemen (MAK). Hautarzt.

[19] Rabe E, Pannier-Fischer F, Bromen K (2003). Bonner Venenstudie der Deutschen Gesellschaft für Phlebologie. Phlebologie.

[20] Schäfer I, Rustenbach SJ, Radtke M (2011). Epidemiologie der Psoriasis in Deutschland – Auswertung von Sekundärdaten einer gesetzlichen Krankenversicherung. Gesundheitswesen.

[21] Schakel K, Schon MP, Ghoreschi K (2016). Pathogenese der Psoriasis. Hautarzt.

[22] Smith VH, Gee BC (2005). A role for tubular compression in the management of psoriasis. Clin Exp Dermatol.

[23] Strober B, Thaci D, Sofen H (2022). Deucravacitinib versus placebo and apremilast in moderate to severe plaque psoriasis: efficacy and safety results from the 52-week, randomized, double-blinded, phase 3 POETYK PSO-2 trial. J Am Acad Dermatol.

[24] Stücker M, Rabe E (2022). Medizinische Kompressionsstrümpfe bei chronischen venösen Erkrankungen und Lymphödem. Dermatologie.

[25] Wakamatsu K, Naniwa K, Hagiya Y (2010). Psoriasis verrucosa. J Dermatol.

[26] Wan J, Wang S, Haynes K (2013). Risk of moderate to advanced kidney disease in patients with psoriasis: population based cohort study. BMJ.

[27] Who Globaler Bericht zur Schuppenflechte, autorisierte deutsche Übersetzung des „Global Report on Psoriasis“ der WHO 2016

